# Three-dimensional innate mobility of the human foot bones under axial loading using biplane X-ray fluoroscopy

**DOI:** 10.1098/rsos.171086

**Published:** 2017-10-18

**Authors:** Kohta Ito, Koh Hosoda, Masahiro Shimizu, Shuhei Ikemoto, Takeo Nagura, Hiroyuki Seki, Masateru Kitashiro, Nobuaki Imanishi, Sadakazu Aiso, Masahiro Jinzaki, Naomichi Ogihara

**Affiliations:** 1 Department of Mechanical Engineering, Faculty of Science and Technology, Keio University, 3-14-1 Hiyoshi, Kohoku-ku, Yokohama 223-8522, Japan; 2 Department of System Innovation, Graduate School of Engineering Science, Osaka University, Toyonaka, Japan; 3 School of Medicine, Keio University, Tokyo, Japan

**Keywords:** foot kinematics, bipedal locomotion, tibio-calcaneal coupling, subtalar joint, midtarsal joint, 2D–3D registration

## Abstract

The anatomical design of the human foot is considered to facilitate generation of bipedal walking. However, how the morphology and structure of the human foot actually contribute to generation of bipedal walking remains unclear. In the present study, we investigated the three-dimensional kinematics of the foot bones under a weight-bearing condition using cadaver specimens, to characterize the innate mobility of the human foot inherently prescribed in its morphology and structure. Five cadaver feet were axially loaded up to 588 N (60 kgf), and radiographic images were captured using a biplane X-ray fluoroscopy system. The present study demonstrated that the talus is medioinferiorly translated and internally rotated as the calcaneus is everted owing to axial loading, causing internal rotation of the tibia and flattening of the medial longitudinal arch in the foot. Furthermore, as the talus is internally rotated, the talar head moves medially with respect to the navicular, inducing external rotation of the navicular and metatarsals. Under axial loading, the cuboid is everted simultaneously with the calcaneus owing to the osseous locking mechanism in the calcaneocuboid joint. Such detailed descriptions about the innate mobility of the human foot will contribute to clarifying functional adaptation and pathogenic mechanisms of the human foot.

## 1. Introduction

The structure of the human foot is thought to be morphologically adapted for the generation of efficient and stable bipedal walking compared to that of non-human primates [1–3]. For example, the human foot uniquely possesses a longitudinal arch that can compress and recoil, allowing the mechanical energy to be stored in the form of elastic energy and successively released during each foot contact [4–6]. The human foot is also known to possess the so-called windlass mechanism in which the prominent plantar aponeurosis is stretched as the metatarsophalangeal joint is dorsiflexed in the late stance phase, in order to restrict and stiffen the midtarsal joints for effective propulsion at the toe-off [7,8]. The stiffening of the midfoot at the late stance phase is also suggested to occur owing to the inversion of the subtalar joint in the late stance phase that changes the directions of the rotational axes of the calcaneocuboid and talonavicular joints and hence restricts the mobility of the midtarsal joints (midtarsal locking mechanism [9,10]). Conversely, when the load is applied in the early stance phase, the subtalar joint is everted so that the midtarsal joint becomes more flexible to allow the foot to accommodate an irregular surface [11] owing to the synergistic movement of the midtarsal joints [12]. However, details of the morphofunctional mechanisms of the structure of the human foot that were possibly acquired in the course of human evolution as the adaptation to habitual bipedalism remain unclear, mainly owing to the difficulties associated with the direct measurement of the foot bones *in vivo*.

To clarify the morphofunctional mechanisms of the human foot, efforts have been made to directly capture the three-dimensional (3D) movements of the foot bones during human walking, as kinematic data based on surface markers are less reliable owing to skin movement artefact [13,14]. Previous studies used bone pins to directly measure the 3D translational and rotational displacements of the foot bones *in vivo* during walking [14–18] and running [19–21]. More recently, single and biplane fluoroscopy were used to quantify the 3D movements of the bones *in vivo* during walking [22–29] as these methods are direct, but less invasive ways to capture the bone movements. In biplane fluoroscopy, the 3D surface models of the foot bones reconstructed from medical images are matched to the fluoroscopic images to capture the 3D foot bone movements. These previous studies successfully demonstrated that there are some stereotypical patterns in midtalar joint movements occurring in the foot during walking. For example, it was found that the talonavicular joint exhibited the greatest mobility and it moved synchronously with the subtalar and calcaneocuboid joints in a stereotypical manner during walking [18,28].

Owing to these efforts, researchers now have a better picture of how the foot bones are spatially translated and oriented during human walking. However, such observed foot bone movements during walking were generated as a consequence of the neural control of forces generated by the extrinsic and intrinsic muscles of the foot. Therefore, it is actually difficult to infer the innate mobility of the foot and how the morphology and structure of the human foot are functionally adapted to bipedal walking just from the *in vivo* kinematics of the foot bones during walking. On the other hand, if we use cadaver feet, such factors can be better controlled, possibly leading to a deeper understanding of the innate patterns of the foot bone movements that are inherently prescribed in the structure of the human foot, and hence the causal relationship between foot bone kinematics and the functional adaptations of the foot bone morphology and structure for generation of bipedal locomotion.

In the present study, we, therefore, quantified the 3D movements of the nine foot bones, i.e. the calcaneus, talus, navicular, cuboid, and five metatarsal (MT) bones, and tibia under a static weight-bearing condition based on cadaver specimens using biplane fluoroscopy and a model registration technique [30], aiming to clarify the patterns of the foot bone movements that are inherently embedded and prescribed in the anatomical structure of the human foot.

## 2. Material and methods

### 2.1. Specimens

Five fresh frozen cadaver lower legs (average age at death, 80 years; range, 68–90 years; two females and three males; one right foot and four left feet) donated to Keio University School of Medicine were used in this study. The study was performed at the Clinical Anatomy Laboratory in Keio University School of Medicine. An informed consent was obtained from the families of all the donors. The present study was approved by the ethics committee of the School of Medicine and the Faculty of Science and Technology, Keio University. All methods were performed in accordance with the relevant guidelines and regulations.

Prior to the experiment, gross visual inspection and radiographic screening were performed to confirm all specimens to be free of foot and ankle pathologies. The specimens were cut at the middle of the lower leg and the soft tissues, such as muscles, were stripped from the shafts of the tibia and fibula.

### 2.2. Experimental set-up

**Figure 1.**
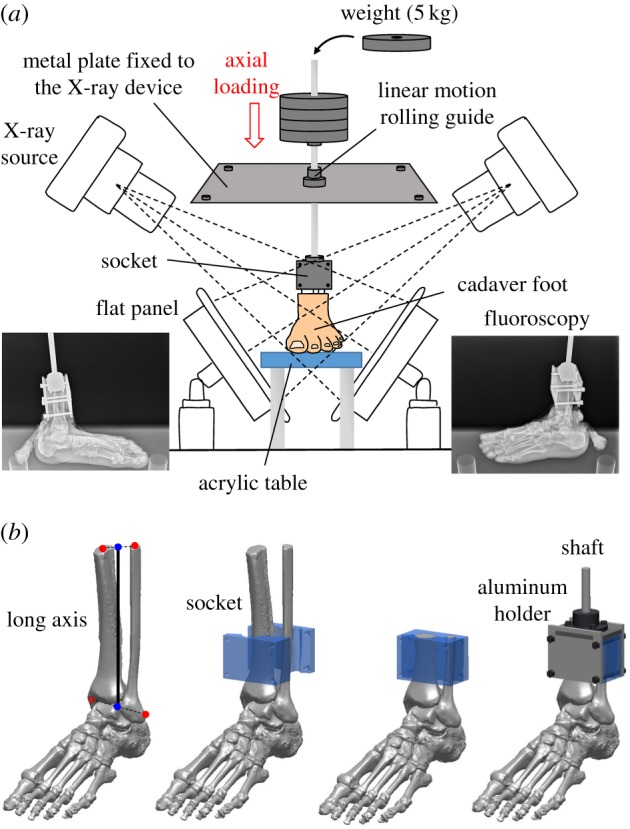
Experimental set-up (*a*) and the socket for the fixation of the lower limb (*b*). Changes in the 3D kinematics of the foot bones during foot–ground contact owing to axial loading were measured using biplane X-ray fluoroscopy and an automatic model registration method (Ito *et al*. [30]). The biplane X-ray fluoroscopy system consists of two X-ray sources and corresponding detector panels positioned in a quasi-orthogonal arrangement. The specimens were fixed to the shaft using a custom-made socket (50 × 70 × 40 mm). The socket was 3D-printed so that it can be placed approximately 80 mm below the proximal end of the tibia and fibula. Its vertical axis was aligned to the long axis of the lower leg (the line connecting the midpoint between the proximal ends of the tibia and fibula (the centroids of the bone sections) and the midpoint between the lateral and medial malleolus). The tibia and fibula were then sandwiched by the anterior and posterior moulds, cut at the upper surface of the socket to coincide the bone sections with the socket, and tightly screwed to an aluminium holder that was connected in line with the shaft.

In the present study, cadaver feet were loaded vertically by putting weight on the shaft connected to the foot, and the 3D movements of the foot bones and tibia owing to the vertical compression were quantified using biplane fluoroscopy (figure 1*a*). The proximal end of the tibia and fibula was fixed to a shaft using a custom-made socket (a 3D-printed mould of the proximal end of the amputated tibia and fibula, 50 × 70 × 40 mm) so that the lower leg and the shaft were aligned on the same line. Specifically, the socket (mould) was 3D-printed so that it can be placed approximately 80 mm below the proximal end of the tibia and fibula. Its vertical axis was aligned to the long axis of the lower leg (the line connecting the midpoint between the proximal ends of the tibia and fibula (the centroids of the bone sections) and the midpoint between the lateral and medial malleolus; figure 1*b*). The tibia and fibula were then sandwiched by the anterior and posterior moulds, cut at the upper surface of the socket to coincide the bone sections with the socket, and tightly screwed to an aluminium holder that was connected in line with the shaft (figure 1*b*).

The shaft goes through a linear motion rolling guide; therefore, the shaft can only move along and rotate around the vertical axis. The specimens were loaded axially by putting 5 kg weights, which were corrected using clay, on the shaft up to 60 kg (588 N). We did not apply forces to the muscle tendons in order to observe innate pure bony movements just produced by the morphology and structure of the human foot.

In some previous cadaveric studies [31,32], ball-bearing plates were placed under the foot specimens to allow abducting movement. Since we intend to simulate actual mechanical interaction of the foot with the ground, a thin rubber sheet was placed on the acrylic table so that the feet did not slip during axial loading (figure 1). Instead, the shaft can freely rotate because of the linear motion rolling guide, allowing unrestrained tibial rotation occurring owing to the kinematic coupling of the calcaneal inversion/eversion and the tibial rotation [33,34]. However, anteroposterior and mediolateral movement of the talus during axial loading might possibly be constrained because of the horizontally restricted movement of the shaft. In the present study, therefore, we used a socket made of rubber-like polymer material (mixture of Tango+ and VeroWhite; shore A hardness = 40) using Object 260 Connex (Stratasys, Eden Prairie, MI, USA), so that the socket permits slight translational and orientational movements of the tibia, allowing physiological movements of the talus during the weight-bearing condition.

### 2.3. Biplane X-ray fluoroscopy and two dimensional–three dimensional registration

We used a custom-made X-ray biplane fluoroscopy system to capture the 3D movements of the nine foot bones and tibia. The fluoroscopic images were taken every 10 kg of applied load. After putting the weight on the shaft, we waited for at least 30 s before obtaining the images to allow the foot bones to settle after loading. Measurement was taken once for each foot. The system consists of two pairs of X-ray sources and flat panels positioned in a quasi-orthogonal arrangement (See Ito *et al*. [30] for details of the system). To reconstruct 3D movements of the foot bones and tibia from the two-dimensional (2D) images, spatial calibration of the biplane fluoroscopy system was first conducted based on the direct linear transformation method. To quantify the 3D position and orientation of each foot bone, a model-based matching method was employed. Briefly, 3D surface models of the bones were generated prior to motion measurement based on computed tomography (See Ito *et al*. [30] for details of the 3D model construction), and the bone models were then registered to biplane fluoroscopic images to maximize similarity measures between occluding contours of the bone surface models with edge-enhanced fluoroscopic images, while avoiding mutual penetration of bones [30]. For the optimization, we used a quasi-Newton method, and this calculation was conducted with custom-made software using Microsoft Visual Studio C++ 2010. The accuracy of the bone registration was approximately 0.3 mm and 0.3° [30].

### 2.4. Quantification of bone movements

**Figure 2.**
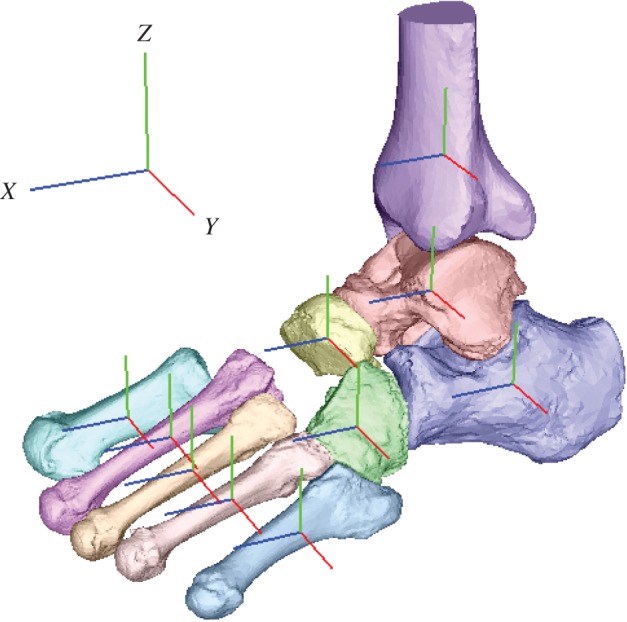
Definition of the coordinate system. A bone-fixed local coordinate system was defined such that the three orthonormal axes (xyz) of the local coordinate system at the neutral posture were aligned with the XYZ axes of the global coordinate system. The x-axis (blue), y-axis (red) and z-axis (green) roughly pointed anteriorly, medially and superiorly, respectively.

To quantify the 3D bone kinematics, a bone-fixed local coordinate system was defined such that the three orthonormal axes (xyz) of the local coordinate system at the neutral posture were aligned with the XYZ axes of the global coordinate system (figure 2). The X, Y and Z axes roughly correspond to inversion–eversion (adduction–abduction for the tibia), plantarflexion–dorsiflexion, and internal/external rotation axes. The origin of the bone coordinate system was defined to be at the centroid of the corresponding bone. The neutral posture (zero loading condition) was defined as the posture when only the vertical shaft (3.3 kg) was attached to the specimen. In the present study, the change in the position and orientation of the bone from the neutral posture was quantified. We used y-x-z Euler angles to describe the orientations of a bone as in Nester *et al*. [14] and Lundgren *et al*. [18] The rotational angles around the y, x and z-axes represent plantarflexion–dorsiflexion, inversion–eversion and internal–external rotation, respectively. Bone-to-bone angles of the subtalar (ST), tabnavicular (TN) and calcaneocuboid (CC) joints were defined as the motion of the distal bone coordinate system with respect to the proximal bone coordinate system. The relative orientation between the navicular and the 1MT (1MT–NAV) was also quantified in the same manner. The mean and standard deviation across five specimens were calculated.

Furthermore, to quantify macroscopic deformation of the cadaver feet owing to axial loading, we measured the changes in the navicular height (the vertical position of the navicular tuberosity from the floor), foot length (the distance between the most posterior point of the calcaneal tuberosity and the most distal point of the medial edge of the 1MT head articular surface) and foot width (the distance between the most distal points of the medial edge of the 1MT and the lateral edge of the 5MT head articular surfaces). The mean and standard deviation across five specimens were calculated.

### 2.5. Statistical methods

The changes in 3D positions and orientations of the foot bones and tibia owing to axial loading (588 N) were evaluated statistically. Specifically, Wilcoxon signed-rank test was performed using SPSS Statistics 21 (IBM, Chicago, IL, USA) to assess that the mean translational and angular displacements across five specimens were significantly different from zero when 588 N of axial load was applied to the foot. The changes of foot dimensions were also evaluated.

## 3. Results

**Figure 3.**
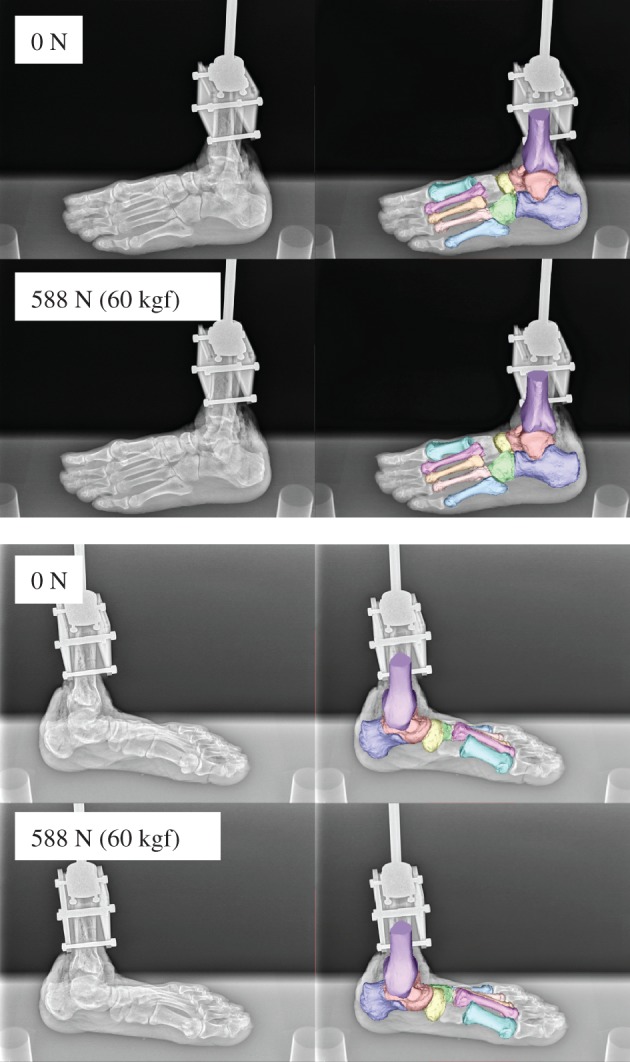
Biplane X-ray fluoroscopic images and corresponding registration results.

**Figure 4.**
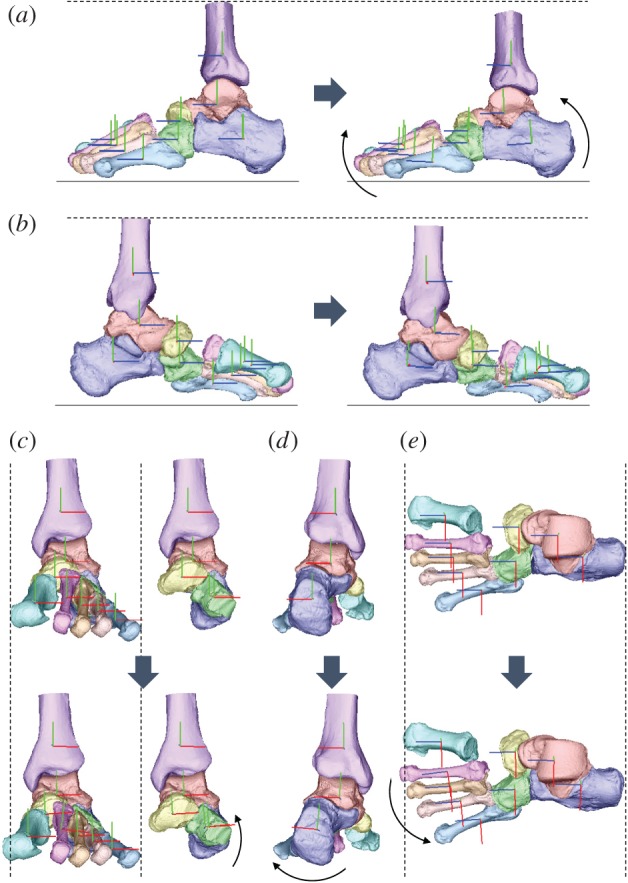
Reconstructed three-dimensional foot bone movement under the axial loading condition at 588 N ((*a*) lateral view, (*b*) medial view, (*c*) anterior view with and without metatarsals, (*d*) posterior view, (*e*) dorsal view).

The 3D skeletal movements of the tibia and foot bones under static axial loading in one representative specimen are illustrated in figures 3 and 4. Movements of the foot bones were successfully reconstructed to match with corresponding fluoroscopic images, as shown in figure 3. Figure 4 compares the 3D foot bones before (0 N) and after the axial load (588 N) was applied.

**Figure 5.**
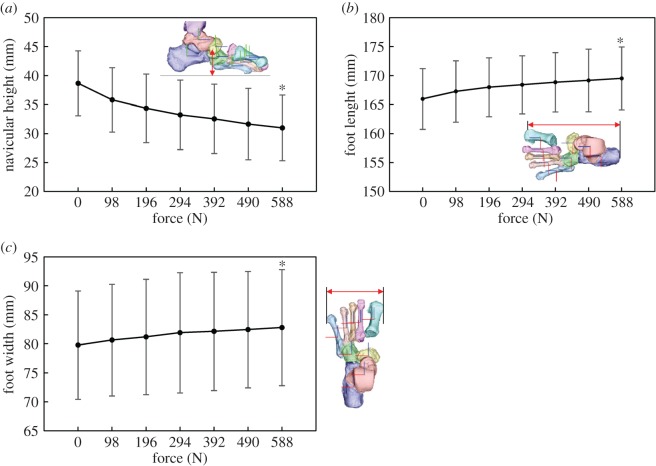
Changes in the foot dimensions during axial loading. (*a*) Navicular height, (*b*) foot length, (*c*) foot width. Means and standard deviations across five specimens were illustrated by markers and error bars, respectively. The asterisk indicates that the mean changes of the foot dimensions at 588 N are significantly different from zero (*p* < 0.05).

Figure 5 shows the changes in the foot dimensions with increasing axial loading. The navicular height decreased, while the foot length increased owing to axial loading. However, the foot width did not change largely. The foot dimensions of each specimen before and after the axial load shown in figure 5 are provided in the electronic supplementary material.

**Figure 6.**
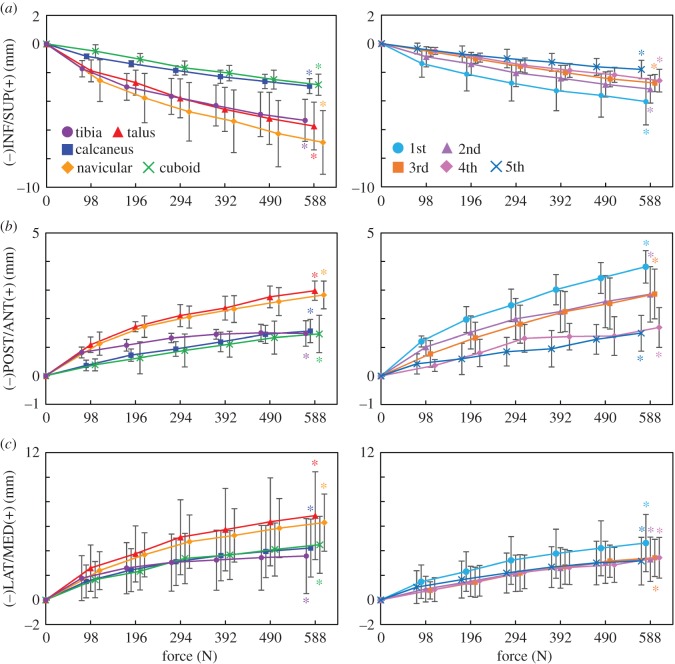
Tri-axial translational displacements of the foot bones in the superoinferior (*a*), anteroposterior (*b*) and mediolateral (*c*) directions during axial loading. The changes in the displacements from the neutral posture were quantified. Means and standard deviations across five specimens were illustrated by markers and error bars, respectively. The values are positive for superior, anterior and medial translations, respectively. The asterisks indicate that the mean translational displacements at 588 N are significantly different from zero (*p *< 0.05).

Figure 6 shows the translational movements of the tibia, tarsal bones and MTs with increasing axial loading. All bones were translated inferiorly, but the vertical translation was larger for the tibia, talus and navicular than for the calcaneus and cuboid, indicating that the medial side of the foot was more flattened than the lateral side (figure 6*a*). The same is true for the MTs; the medial 1MT exhibited the largest vertical displacement, and the displacement of the other MTs was decreased laterally with distance from the 1MT. In addition, with increasing axial loading, all the foot bones moved anteriorly (figure 6*b*). The anterior displacement was larger in the talus and navicular than in the calcaneus and cuboid. The largest anterior movement was observed in the 1MT, and the displacement of other MTs decreased laterally with distance from the 1MT. Furthermore, the foot bones were observed to move medially (figure 6*c*), but the medial translation of the talus and navicular was larger than that of the calcaneus and cuboid, indicating that the talus slid medially with respect to the calcaneus. The MTs were also translated medially, with the 1MT exhibiting the largest medial translation.

**Figure 7.**
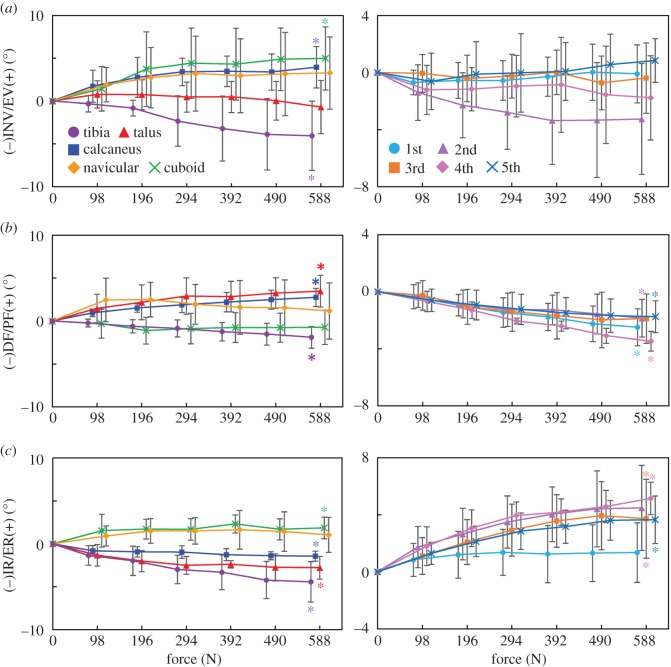
Tri-axial rotations of the foot bones in the coronal (*a*), sagittal (*b*) and transverse (*c*) planes during axial loading. The changes in the displacements from the neutral posture were quantified. Means and standard deviations across five specimens were illustrated by markers and error bars, respectively. The values are positive for eversion, plantarflexion and external rotation. The asterisks indicate that the mean angular displacements at 588 N are significantly different from zero (*p *< 0.05).

Figure 7 shows the rotational movements of foot bones and tibia with increasing axial loading. While the tibia was rotated in the adducting direction in the global coordinate frame, the navicular, calcaneus and cuboid were rotated in the everting direction; no inversion/eversion movement was observed for the talus (figure 7*a*). The MTs were generally inverted, with the 2MT exhibiting the largest inversion and the 1MT and 5MT exhibiting the least inversion with increasing axial loading. The talus and calcaneus were rotated in the plantarflexing direction as the foot flattened, but such rotational movement was not observed for the cuboid (figure 7*b*). On the other hand, all MTs were rotated in the dorsiflexing direction owing to the flattening of the foot. Horizontally, the tibia, talus and calcaneus were all rotated in the direction of internal rotation. However, all distal bones (navicular, cuboid and MTs) were rotated in the direction of external rotation. The 3D position and orientation of each foot bone model before and after the axial load, shown in figures 6 and 7, are provided in the electronic supplementary material.

**Figure 8.**
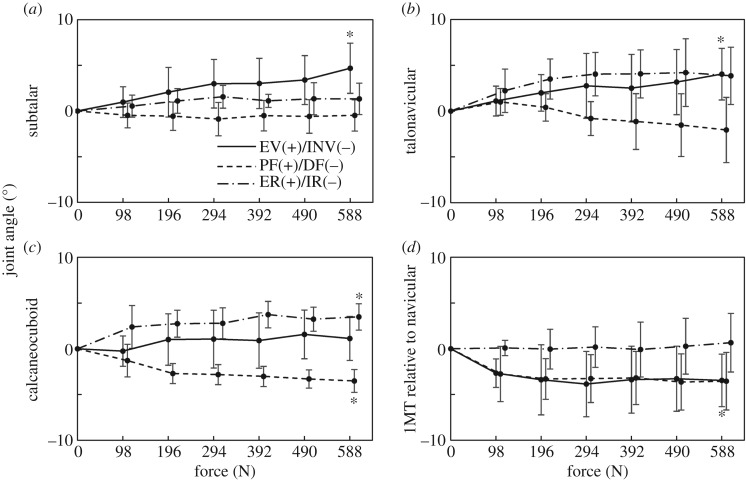
Changes in subtalar (ST) (*a*), talonavicular (TN) (*b*), calcaneocuboid (CC) (*c*) joints and orientation of the first metatarsal with respect to navicular (1MT–NAV) (*d*) with increasing axial loading. The angles are positive for eversion, plantarflexion and external rotation. Means and standard deviations across five specimens were illustrated by markers and error bars, respectively. The asterisks indicate that the mean angular displacements at 588 N are significantly different from zero (*p *< 0.05).

Figure 8 shows the joint angle profiles of the ST, TN and CC joints, as well as 1MT–NAV. In the present study, the ST, TN and CC joints were all everted, dorsiflexed and externally rotated, respectively, with increasing load. The 1MT was dorsiflexed and inverted with respect to the navicular in the present study. The relative joint movements were consistent with the absolute rotations of the foot bones presented in figure 7. The joint angles of each specimen before and after the axial load shown in figure 8 are provided in the electronic supplementary material.

## 4. Discussion

We found that the talus is medially translated and internally rotated as the calcaneus is everted during axial loading in the human foot. Since the talus is articulated superomedially with respect to the calcaneus, the eversion of the calcaneus is not surprising and such calcaneal eversion after heel contact has actually been documented in both human walking [35–37] and running [21,38]. However, our fluoroscopy study demonstrated that the talus slid down the ST articular surfaces of the calcaneus as it inclined owing to the eversion of the calcaneus. In addition, the talus was simultaneously internally rotated, possibly because of the shape and geometry of the ST and TN joints. Furthermore, the tibia was also internally rotated in conjunction with the talus. Such observed kinematic coupling between the calcaneal eversion and internal tibial rotation has been documented previously [11,33,34,39]. However, the present study quantitatively described to our knowledge, for the first time, if not all, the detailed kinematic mechanism underlying the tibio-calcaneal coupling [33,34] in the human foot. Since the feet of chimpanzees and other apes are more inverted with respect to the tibia [40], inversion, but not eversion, would occur during foot–ground contact [41,42]. Therefore, this morphologically embedded tibio-calcaneal kinematic coupling is probably one of the derived morphological traits that are unique in the human foot that possibly facilitates bipedal locomotion. The same experiment should be conducted using feet of apes in future studies to confirm this hypothesis.

We also identified the innate movements of the MT bones associated with the flattening of the medial longitudinal arch of the human foot. Owing to the internal rotation of the talus with respect to the calcaneus, the talar head moved medially. Therefore, the compressive force between the talar head and navicular was applied more medially, so that the navicular and 2–5MTs were externally rotated, while the 1MT was relatively translated more anteriorly. The present study demonstrated that the characteristic movement of the talus could possibly induce the external rotation and anterior translation of the MTs with increasing axial loading. During walking, the vertical ground reaction moment is applied to the foot in the direction of external rotation in the first half and in the direction of internal rotation in the second half of the stance phase, respectively [43–45]. The structurally embedded capacity of the human foot to generate tibial internal rotation and MT's external rotation may possibly facilitate compensation of the moment generated around the vertical axis of the body during walking owing to trunk rotation and leg swing. Elucidating the possible contribution of the foot structure on generation of a vertical free moment for stable bipedal locomotion would be an important area of future studies.

The present study found that the cuboid everted concomitantly with the eversion of the calcaneus; hence, the change in the inversion/eversion angle of the CC joint was very small during axial loading. This is possibly owing to a locking mechanism of the CC joint. The human cuboid possesses a prominent medial calcaneal process that articulates with a deep concavity on the distomedial surface of the calcaneus [46]. Therefore, the human CC joint is structurally more constrained than those of the apes, whose midfoot regions are known to be more mobile than those of humans [47]. However, the midtarsal locking mechanism [48] actually explains the relative stiffening of the midtarsal region owing to inversion of the calcaneus with respect to the talus. In the present study, the foot bone kinematics under axial loading corresponding to the former half of the stance phase was investigated, but not the latter half of the stance phase when the foot acts as an effective lever for generation of propulsive force. Recent studies suggested the CC joint is far more mobile than previously believed [49]. We aim to investigate the innate mobility of the foot bones during push-off in future studies to clarify the innate mobility of the CC joint in the human foot and in the feet of African apes for comparative understanding of the derived morphofunctional features embedded in the human foot [50,51].

We observed that the 2MT was largely inverted under axial loading, but the inversion/eversion movement of the 1MT was almost negligible, although the navicular was everted. This indicates that the metatarso-cuneiform joints were actually quite mobile in the axially loaded cadaver feet with no muscle contractions. Previous cadaver studies suggested that the 1MT is everted owing to the traction of the peroneus longus tendon, and this eversion of the 1MT brings the tarsometatarsal joints into a closely packed position to stabilize the forefoot [52,53]. However, when no muscles are retracted, as in this study, the 1MT is found not to be everted, allowing the 2MT to rotate in the inverting direction during axial loading. The 2MT base is generally considered stable in the human foot [54], because the 2MT base is sandwiched by the medial and lateral cuneiforms, but the present study demonstrated that the 2MT is actually mobile in the inverting direction when no foot muscle tractions were present. This implies that if foot muscles, such as the peroneus longus, are weakened possibly because of ageing, the 2MT starts to rotate in the inverting direction under axial loading, and the metatarso-cuneiform joints would start to be loosened, possibly leading to the onset of foot pathologies such as hallux valgus [55]. In the present study, we did not measure the kinematics of the cuneiforms during axial loading, but such information is crucial for understanding the causative factors in the formation of foot pathologies.

In conclusion, the present study successfully establishes the innate whole foot bone mobility under axial loading that is embedded in the morphology and structure of the human foot possibly evolved to facilitate the generation of efficient and robust bipedal locomotion. Such detailed descriptions about the innate mobility of the human foot possibly contribute to clarifying functional adaptation and pathogenic mechanisms of the human foot. However, the present study has some limitations. First, we did not attempt to quantify the movements of the three cuneiforms during axial loading, because these small bones have relatively few characteristic morphological features and are heavily overlapped with one another on the fluoroscopic images, making contour matching very difficult. However, the cuneiforms could probably be matched successfully to the fluoroscopic images if the relative arrangement of the two sets of the X-ray source and the flat panels was adjusted. Second, the cadaver lower legs used in the present study were all from elderly individuals, as in other cadaver studies, but it has been reported that the viscoelastic properties of the plantar soft tissue [56] and joint mobility [57] could change with age, indicating that the identified innate foot mobility during axial loading might be different in non-elderly specimens. Although obtaining young specimens is generally difficult, possible age differences in the foot mobility should be investigated when chances arise. Finally, although we successfully clarified the innate mobility of the human foot bones, the present study cannot assess whether the bone movements occur owing to osseous morphology of the bones and articular surfaces, and/or configuration and distribution of soft tissues such as ligament and adipose. Possible contribution of the soft tissue morphology to generation of human bipedal walking has generally been overlooked and is not well understood. However, this should also be investigated in future studies.

## Supplementary Material

Data set for figure 5-8

## Data accessibility

The datasets supporting this article have been uploaded as part of the electronic supplementary material.
